# A Case of Metastatic Melanoma Refractory to Immunotherapy: Is Cytotoxic Chemotherapy Still an Effective Option?

**DOI:** 10.7759/cureus.104178

**Published:** 2026-02-24

**Authors:** Maleeha Anwar, Ricky Frazer, G.J Melendez-Torres, Veena Mariam Joseph, Charlotte Bennett

**Affiliations:** 1 Medical Oncology, Velindre Cancer Centre, Cardiff, GBR; 2 Immuno-Oncology, Velindre Cancer Centre, Cardiff, GBR; 3 Clinical and Social Epidemiology, University of Exeter, Exeter, GBR; 4 Oncoradiology, Velindre Cancer Centre, Cardiff, GBR; 5 Medicine, Royal Gwent Hospital, Newport, GBR

**Keywords:** chemotherapy sensitive mucosal melanoma, dacarbazine chemotherapy in melanoma, immunotherapy refractory melanoma, mucosal malignant melanoma, sinonasal mucosal melanoma

## Abstract

Metastatic melanoma is traditionally associated with poor responses to conventional cytotoxic chemotherapy. Although there has been a shift in paradigm in melanoma treatment in the last few decades with the use of targeted treatment and immunotherapy, there is a significant subset of patients who are treatment refractory. Interestingly, some of these patients may derive meaningful benefit from cytotoxic chemotherapy. We present the report of a case of a 63-year-old lady with *BRAF* wild type, locally advanced mucosal melanoma who demonstrated disease progression on combination immunotherapy with Ipilimumab and nivolumab. She subsequently achieved an exceptional and durable radiological and clinical response to chemotherapy with cisplatin and dacarbazine. This case highlights that, despite advances in personalized medicine and immunotherapy, conventional chemotherapy may still represent a valuable therapeutic option in selected patients, particularly in the refractory setting.

## Introduction

Sinonasal mucous melanoma is a rare malignancy that accounts for 0.5% of all melanomas and 4-7% of all sinonasal malignancies. It exhibits poor prognosis and an unpredictable pattern with local recurrence and distant metastasis. The five-year overall survival is around 30-40% [1].

Surgical resection with clear margins followed by postoperative radiation therapy is the gold standard of treatment [2,3]. In relapsed, unresectable, and metastatic cases, the recommended treatment options are combination immunotherapy with ipilimumab and nivolumab, single-agent immunotherapy, targeted treatments in cases of actionable mutations, or conventional chemotherapy. Although melanoma is known to be chemo-resistant, chemotherapy was the mainstay of treatment before the era of targeted treatment and immunotherapy. The response rate with chemotherapy was modest. The most widely used regimens are dacarbazine-based with objective response rates (ORRs) of 5-20% [4]. The combination of dacarbazine with other agents, especially cisplatin, produced better results than dacarbazine alone in terms of ORR and progression-free survival (PFS), but not overall survival (OS) [5].

Immunotherapy and targeted treatments have revolutionised the treatment of cutaneous melanoma. Five-year survival rates for single-agent programmed cell death protein 1 (PD-1) inhibitors of 34-44% in stage IV disease [5,6] and 52% when combined with the cytotoxic T-lymphocyte-associated protein 4 (CTLA-4) inhibitor ipilimumab [6]. Therefore, immune checkpoint inhibitors with programmed-death ligand 1 (PD-L1) inhibitor, either alone, with pembrolizumab, or in combination with CTLA 4 inhibitor Ipilimumab is the recommended treatment by European Society for Medical Oncology (ESMO), American Society of Clinical Oncology (ASCO), and National Comprehensive Cancer Network [7-9].

In 40-50% of patients with cutaneous melanoma, a targetable mutation in *BRAF* is identified. They are targetable by BRAF inhibitors (BRAFi), including vemurafenib, dabrafenib, and encorafenib. When used in combination with MEK inhibitors (MEKi) (cobimetinib, trametinib, or binimetinib), which target downstream kinases in the MAPK pathway, five-year survival for BRAF inhibitors is 31-35% [10-12]. However, the incidence of *BRAF* mutation in mucosal melanoma is much lower and is quoted to be around 3-15% [13].

The representation of patients with mucosal melanoma in melanoma clinical trials is dominated by the cutaneous melanoma group. Patients with mucosal melanoma are fewer, and the site of origin is not identified, which makes it challenging to interpret the data for patients with mucosal melanoma. However, subgroup analysis has demonstrated a lower level of efficacy with treatment compared with cutaneous melanoma, with an ORR of combination immunotherapy of 37% [14].

There are currently no guidelines for the use of cytotoxic chemotherapy in melanoma due to the historically chemoresistant nature of this disease. Consequently, the use of chemotherapy in melanoma is limited across the United Kingdom, with most centres no longer routinely using cytotoxic agents. We present a case highlighting that, in patients with melanoma who do not respond to immunotherapy and targeted treatments, chemotherapy may still represent a potentially effective treatment option.

## Case presentation

In June 2022, a woman in her early 60s was referred to the oncology clinic with recurrence of melanoma in the right nasal cavity. She had no other significant past medical history and was not on any regular medication. She initially presented six years ago with a history of anosmia and nasal obstruction. This was confirmed through biopsy to be malignant melanoma of the right nasal cavity. She underwent trans nasal endoscopic anterior skull base resection in June 2016. Surgical margins were clear. Her tumour was *BRAF* wild type, and KIT mutation analysis was unsuccessful due to insufficient neoplastic cells in the tissue sample. Surgical resection was followed by adjuvant radiotherapy 60Gy in 30 fractions over 40 days, which she completed in September 2016.

The patient remained under active surveillance with both ENT and oncology follow-up and has had serial scans and nasoendoscopies with no evidence of recurrence. However, in May 2020, she presented to the Accident and Emergency (A&E) department with a nosebleed; the bleeding point was cauterised, and epistaxis settled. Her follow-up appointment with ENT showed no recurrence on nasoendoscopy. The following year, she had intermittent epistaxis; examination revealed likely lisch vessels. She continued to have epistaxis, leading to A&E visits on two occasions in April 2022.

**Figure 1:**
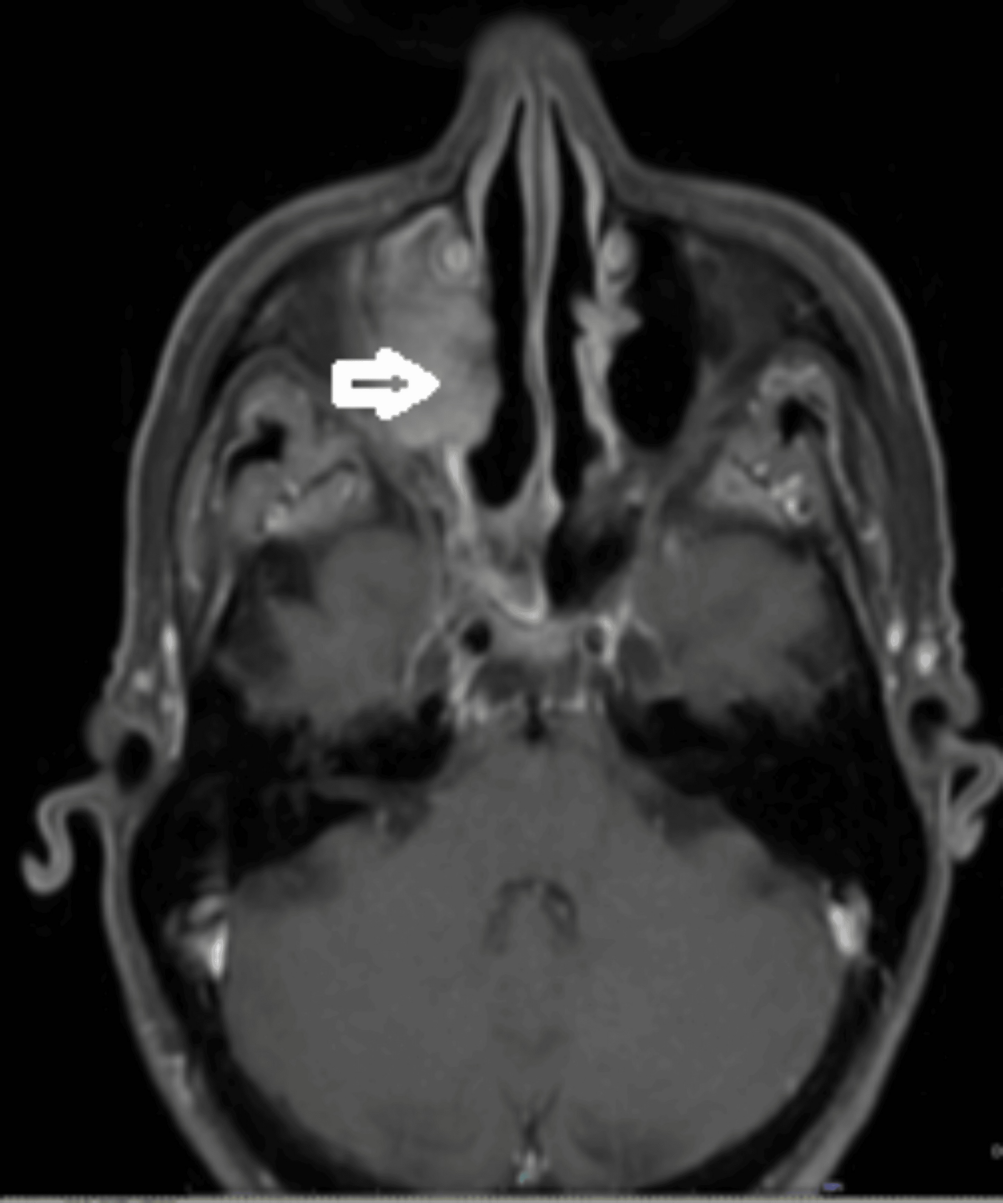
MRI sinus showing tumour extending into the nasal cavity.

**Figure 2:**
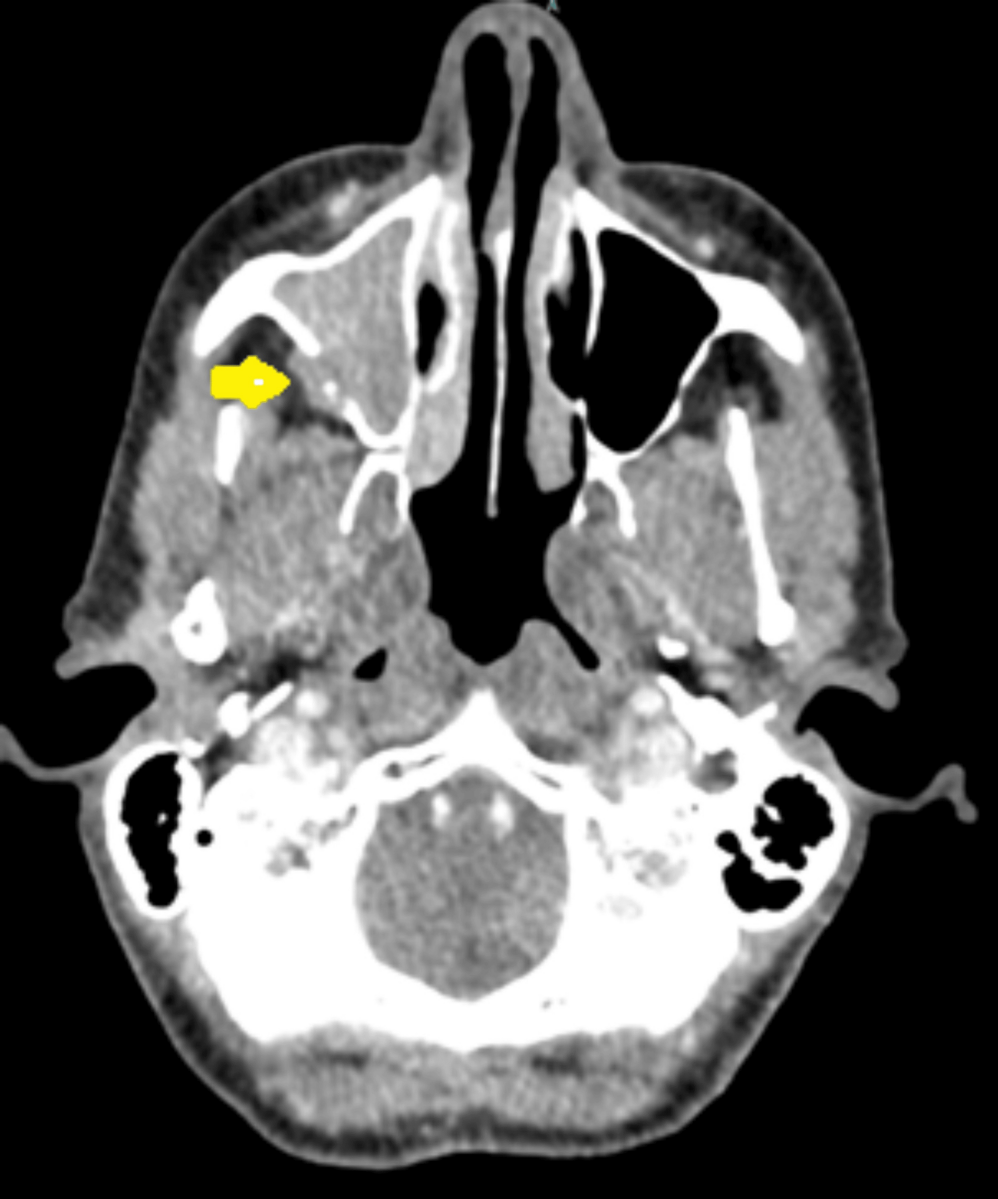
Contrast-enhanced CT in May 2022 (Axial view) Recurrent tumour filling the right maxillary antrum breaching the posterior wall and extending into the retro maxillary fat (yellow arrow).

**Figure 3:**
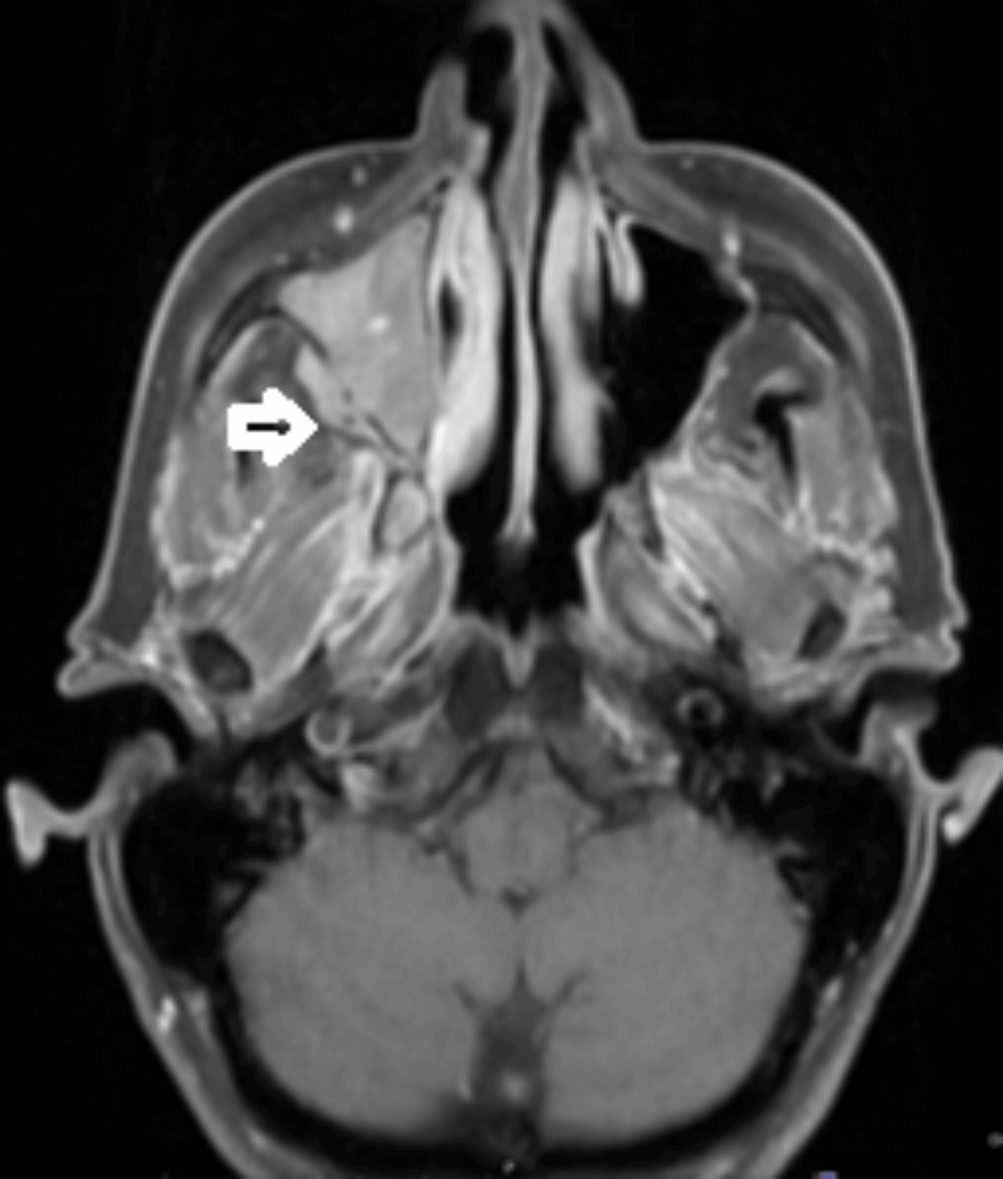
Post-gadolinium T1 weighted MRI Head in June 2022 (axial view) Slight enlargement of the intramaxillary recurrent tumour, which is also extending to the retromaxillary fat.

She was later seen in the ENT clinic, and a large granular nasal polyp was removed in April 2022. Unfortunately, histology confirmed this to be a recurrence of *BRAF* wild-type melanoma. She subsequently had a staging CT of head, neck, thorax, abdomen, and pelvis in May 2022, which showed recurrent melanoma occupying the right maxillary antrum, which had breached the lateral wall of the antrum and bulged into the fat of the infratemporal fossa (Figure 1). There was no evidence of metastatic disease on her scans. She had an MRI of the head and sinuses in June 2022, which showed a tumour filling the right maxillary antrum extending into the nasal cavity, retro maxillary fat, pterygopalatine fossa, and inferior orbital fissure. This had increased slightly in size since the CT in May (Figures 2, 3).

**Figure 4:**
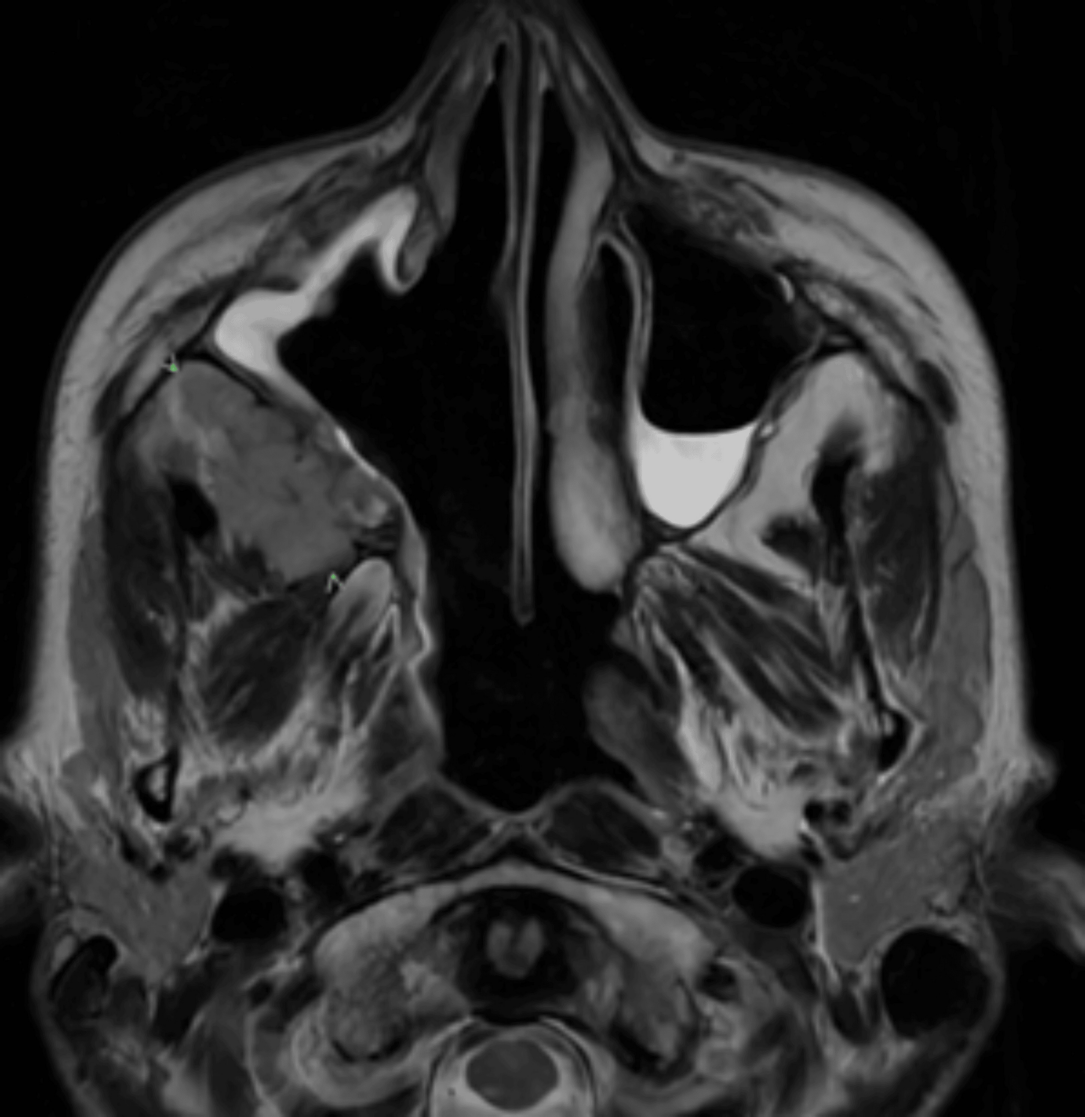
T2-weighted MRI Head and Sinus September 2022 Progressive malignancy with enlarging tumour in the infratemporal fossa (green arrow heads), infiltration into the temporalis, lateral pterygoid and the orbit. Post-surgical changes in the right nasal cavity and medial wall of maxillary sinus can be seen.

**Figure 5:**
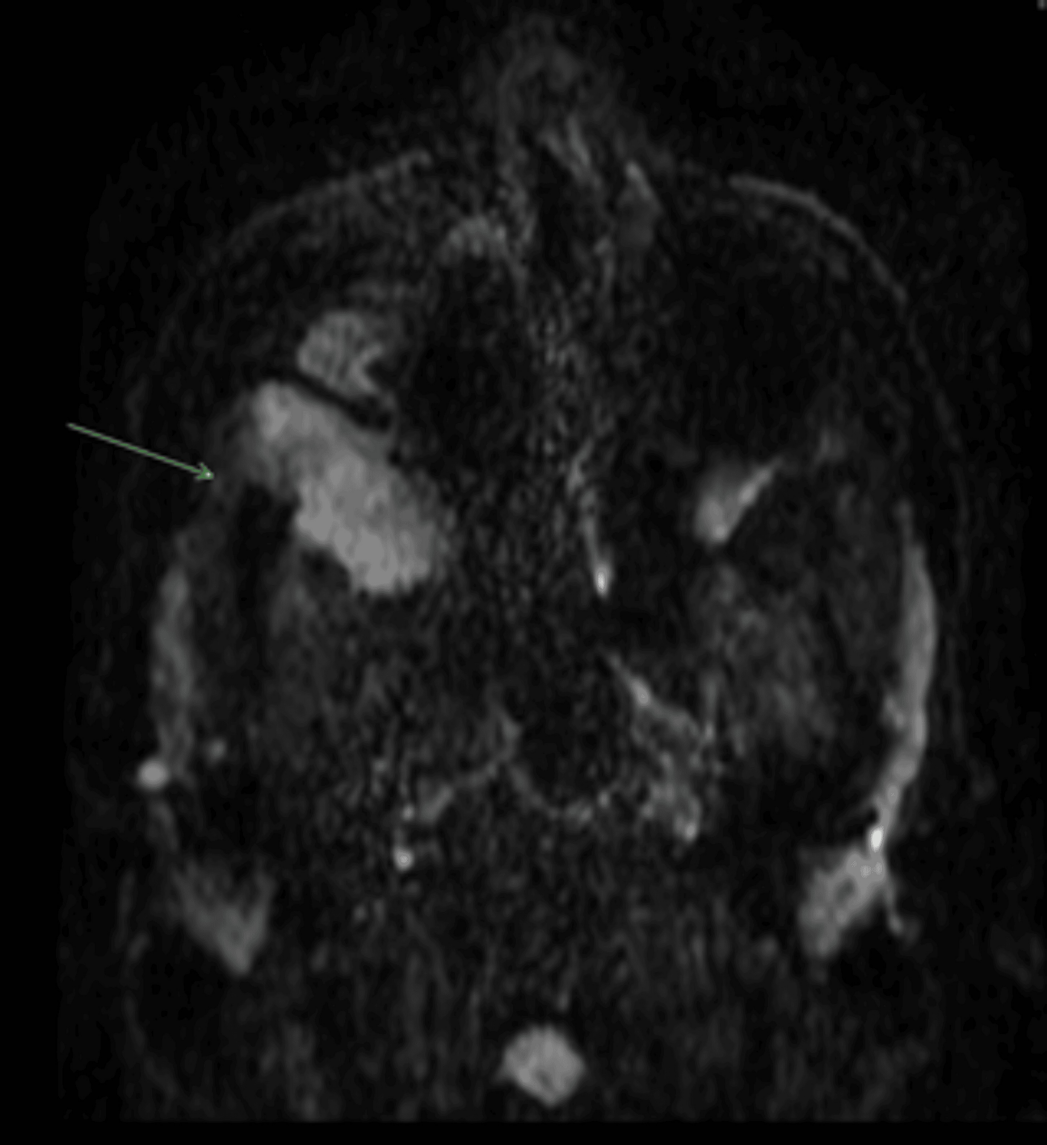
High b value diffusion weighted imaging (DWI) MRI Head and Sinus September 2022 Abnormal restricted diffusion within the tumour. Corresponding apparent diffusion co-efficient (ADC) map show low intralesional ADC values.

The patient underwent non-curative nasoendoscopic debulking in June and made a good recovery. She was commenced on combination immunotherapy with ipilimumab and nivolumab in July 2022. She had her fourth cycle in September, followed by assessment scans. She received the first cycle of maintenance nivolumab in October 2022 while waiting for scan results. MRI showed progressive disease with a significant increase in the size of the tumour. It was now infiltrating the temporalis, the infratemporal fossa, the lateral pterygoid, and the orbit (Figures 4, 5).

**Figure 6:**
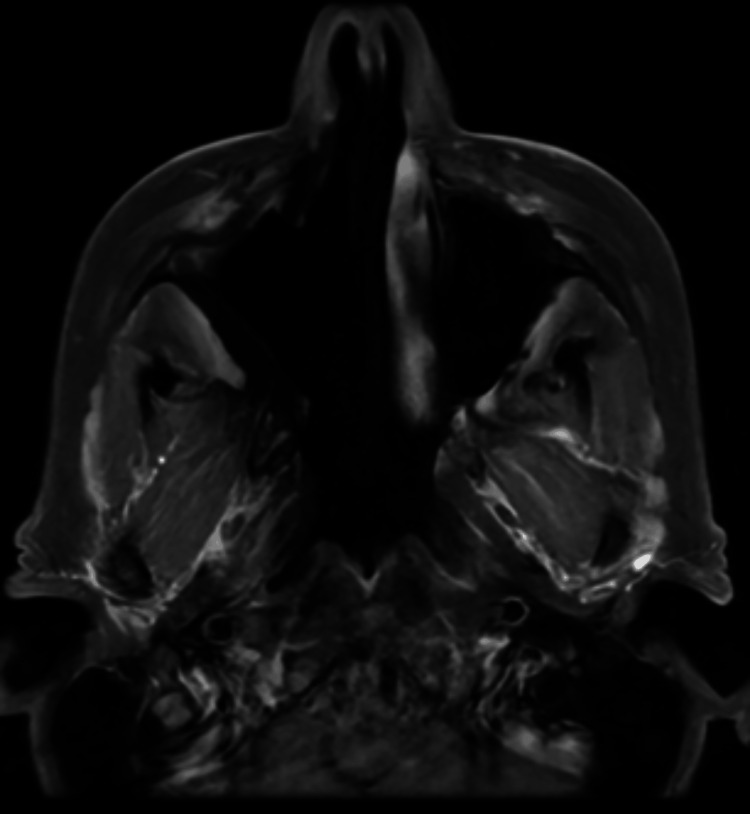
Post-gadolinium T1 weighted MRI in August 2023 (axial view) Post-treatment inflammation without any residual tumour.

**Figure 7:**
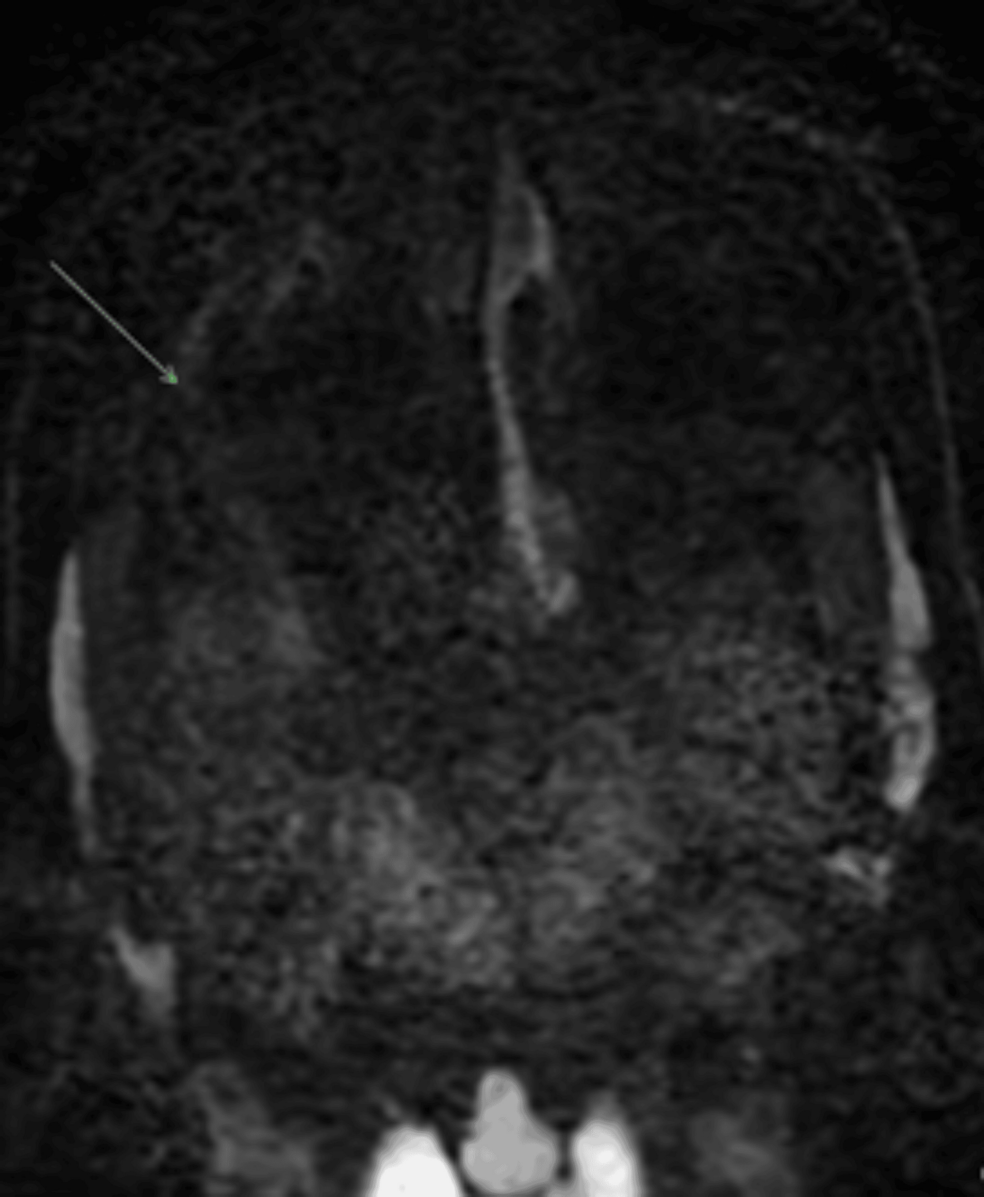
Post-chemotherapy diffusion weighted imaging (DWI) MRI August in 2023 shows normalisation of this area

**Figure 8:**
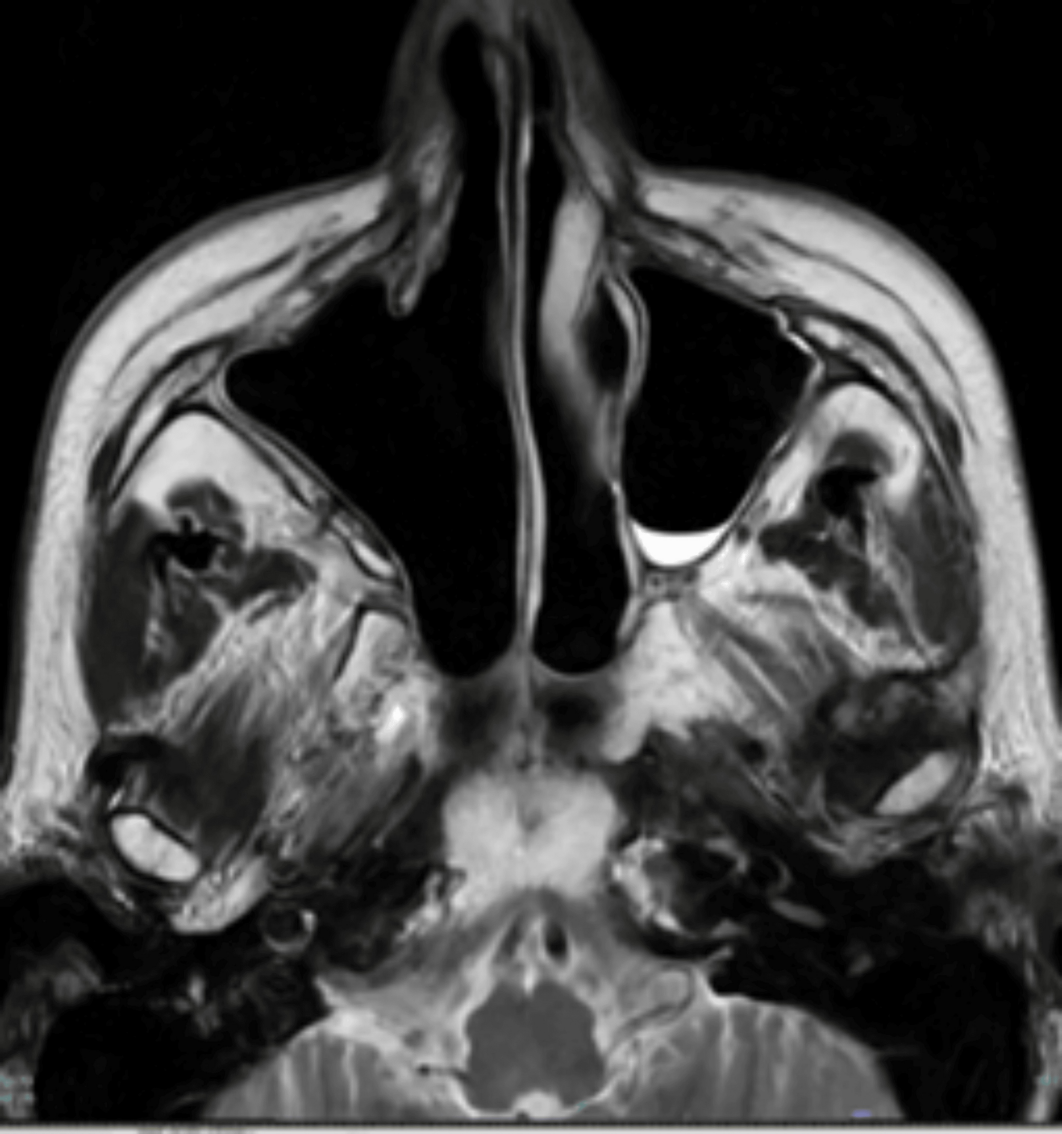
Post-gadolinium T1 weighted MRI in February 2025 (axial view) showing maintained complete local response

Patient consented to undergo second-line treatment with cisplatin and dacarbazine, which started in December 2022. Follow-up CT and MRI in February 2023 showed good partial response with a reduction in the volume of tumour, residual tumour measuring 1.3 x 0.5 cm (2.9 x 1.3 cm before). She received an additional three cycles and follow-up CT and MRI in June 2023 showed stable disease, tumour measuring 1.4 x 0.4 cm (1.3 x 0.5 cm previously). She completed her six cycles of chemotherapy in June 2023. Her next scan in August 2023 showed no evidence of residual disease (Figures 6, 7). She continues to be under active surveillance with three monthly MRIs and CT scans, and is in remission (Figure 8).

## Discussion

In contrast to the recent improvement in the outcome of patients with cutaneous melanoma, the outlook for mucosal melanoma remains poor. There are few clinical trials for only mucosal melanoma, which is largely due to the rarity of this tumour. The representation of mucosal melanoma in any melanoma trial is very small, and response rates to treatment are lower than for cutaneous melanoma.

Chemotherapy combinations have shown efficacy in the Chinese population in a randomized phase II trial of 114 patients with advanced melanoma, in which chemotherapy with carboplatin and paclitaxel with or without the addition of bevacizumab was studied [15]. The study showed significantly longer median PFS and OS with chemotherapy and bevacizumab compared to chemotherapy alone (median PFS 4.8 months vs. 3.0 months and median OS 13.6 vs. 9.0 months).

In addition to this, a multicentre phase II randomized controlled trial of 189 patients with stage II/III disease in China showed significant benefit for both recurrence-free survival (RFS) and OS with adjuvant chemotherapy (temozolomide and cisplatin) compared with observation, high-dose interferon (HDI). After a median follow-up of 26.8 months, median RFS was 5.4 months (observation), 9.4 months (HDI), and 20.8 months (chemotherapy), and median OS was 21.2 months (observation), 40.4 months (HDI), and 48.7 months (chemotherapy) [16].

In a subsequent multicenter phase III randomized controlled trial in China of 204 patients with resected mucosal melanoma, adjuvant chemotherapy with cisplatin and temozolomide led to a significantly lower risk of relapse and distant metastasis compared with HDI. At a median follow-up of 64.8 months, median RFS was 15.5 months with chemotherapy and 9.9 months with HDI. Median distant metastasis-free survival was 19.5 vs. 12.7 months, and OS was 38.2 vs. 33.5 months [17].

These differences in outcomes mentioned above, good response to chemotherapy and the standard cutaneous melanoma approach, must influence future decision-making when choosing treatment, appreciating that the representation of mucosal melanoma in clinical trials is small.

Due to the very poor response to chemotherapy in melanoma, chemotherapy is rarely used across most sites in the United Kingdom. However, the National Institute for Health and Clinical Excellence (NICE) guidelines continue to recommend dacarbazine as a systemic anticancer treatment for unresectable stage III and IV melanoma [18]. Our case highlights that there may still be a subset of melanoma patients where chemotherapy can be utilised and is, in fact, more effective than newer agents. It is vital to recognise this subset of patients to avoid unnecessary toxicity from immunotherapy. In our case, due to limited resources, we were unable to do molecular sequencing to identify deficient DNA repair pathways, which could explain the exceptional response to chemotherapy. There are cases of melanoma that have a better response to conventional chemotherapy, but we need to identify biomarkers that could predict this response. In our case, one explanation could be that the chemotherapy potentiated the immunotherapy, or it was a delayed response, but radiologically and clinically, the degree of growth was not typical for pseudo-progression.

## Conclusions

Mucosal melanoma is a rare form of melanoma that is less responsive to treatments than cutaneous melanoma. Diagnosis can be challenging due to the anatomical location. Even in this era of modern personalised medicine, chemotherapy can still lead to durable outcomes and should be considered as a second-line option for patients with immunotherapy and *BRAF*-targeted therapy refractory melanoma. We need to identify biomarkers to predict response to chemotherapy in melanoma patients.
